# Content of Essential Trace Elements in the Hair of Residents of the Caspian Region of the Republic of Kazakhstan Who Recovered from COVID-19

**DOI:** 10.3390/diagnostics12112734

**Published:** 2022-11-08

**Authors:** Gulnara Batyrova, Zhenisgul Tlegenova, Victoria Kononets, Gulmira Umarova, Yerlan Bazargaliyev, Gulaim Taskozhina, Nurgul Kereyeva, Yeskendir Umarov

**Affiliations:** 1 Department of Laboratory and Visual Diagnostics, West Kazakhstan Marat Ospanov Medical University, Aktobe 030019, Kazakhstan; 2 Department of Internal Diseases No. 2, West Kazakhstan Marat Ospanov Medical University, Aktobe 030019, Kazakhstan; 3 Department of Natural Sciences, West Kazakhstan Marat Ospanov Medical University, Aktobe 030019, Kazakhstan; 4 Department of Evidence-Based Medicine and Scientific Management, West Kazakhstan Marat Ospanov Medical University, Aktobe 030019, Kazakhstan; 5 Department of Internal Diseases No. 1, West Kazakhstan Marat Ospanov Medical University, Aktobe 030019, Kazakhstan; 6 Department of Oncology, West Kazakhstan Marat Ospanov Medical University, Aktobe 030019, Kazakhstan

**Keywords:** Caspian region, COVID-19, essential trace elements, Kazakhstan

## Abstract

This study aimed to investigate the content of essential elements in the hair of unvaccinated residents of the Caspian region who recovered from COVID-19. This cross-sectional study involved 260 unvaccinated permanent residents of Mangistau oblast aged 18–60. The diagnosis and severity of COVID-19 were based on clinical signs and symptoms, laboratory data, R-graph results, and oxygen saturation by the Clinical Protocol of the Ministry of Health of the Republic of Kazakhstan. Inductively coupled plasma mass spectrometry determined the content of trace elements cobalt (Co), chromium (Cr), copper (Cu), iron (Fe), iodine (I), manganese (Mn), selenium (Se), and zinc (Zn). The content of Cr (*p* < 0.05), Cu (*p* < 0.05), Fe (*p* < 0.001), I (*p* < 0.05), Mn (*p* < 0.001), and Zn (*p* < 0.05) in the hair of individuals who had a coronavirus infection was lower than those who did not have this infection. There were significantly higher levels of Cu (*p* < 0.05) in the hair of participants who had moderate or severe COVID-19 compared to those with mild forms. The results of multiple regression analysis showed that in the presence of a COVID-19 infection in a subject’s history, the content of Cr (0.871 (95% CI: 0.811; 0.936)), Cu (0.875 (95% CI: 0.803; 0.955)), Fe (0.745 (95% CI: 0.636; 0.873)), and Mn (0.642 (95%CI: 00.518; 0.795)) decreased in the hair. The data obtained indicate that past COVID-19 infections affect the trace element status of the inhabitants of the Caspian region of Kazakhstan.

## 1. Introduction

The COVID-19 pandemic has highlighted the importance of a well-functioning immune system with significant societal, economic, and health impacts. Along with immutable factors that affect the immune status, such as heredity and belonging to a particular age group, it is affected by changeable factors, which can be adjusted. These include stress, physical fitness, obesity, and nutrient and micronutrient intake [1]. The elemental status of the body is a reflection of the consumption of macro- and trace elements, primarily with food; chemical elements also enter the body in other ways during household and professional activities. There is a definite connection between the state of immunity and elemental status. Reduced intake of certain micronutrients, and the associated decrease in immune status, may cause increased susceptibility to infections, including COVID-19, and lead to a more severe course of the infection [2,3].

Chemical elements, including macro- and trace elements, play a vital role in maintaining the integrity of various physiological and metabolic processes occurring in living organisms, coping with ongoing oxidative stress in body tissues and maintaining sufficient immunity against pathogens. The human body has a complex system of control and regulation of the levels of crucial macro- and trace elements. Abnormal levels of these can develop when there is a malfunction in the body or when they are in excess or deficiency in dietary sources [4].

The role of essential trace elements such as zinc, copper, and iron in supporting the immune status is well studied. Selenium, magnesium, and other trace elements also play a particular role in supporting the immune response. Cell-mediated processes of innate immunity, such as proliferation, differentiation, the performance of certain functions, cell movement, and the ability to produce optimal amounts of free radicals, depend on adequate amounts of iron, zinc, copper, selenium, and magnesium. Zinc, iron, and selenium are involved in activating the complement system and releasing pro-inflammatory cytokines. Iron, zinc, and copper are involved in regulating the inflammatory response. The self-defense of immune cells requires the trace elements zinc, iron, magnesium, copper, and selenium through antioxidant mechanisms [5]. A deficiency of these micronutrients impairs many aspects of both innate and adaptive immunity and increases susceptibility to infections [3,5,6]. The trace element status may be associated with susceptibility to the SARS-CoV-2 infection, the severity and duration of COVID-19, and the development of complications of this infection [7,8,9]. Trace elements are indispensable for forming an immune response to vaccination, and monitoring trace element status can predict the severity of the disease and mortality [10]. Ensuring optimal trace element status can improve the vaccine response to COVID-19 [6,11].

According to Galmes et al., iron intake correlates with lower morbidity and mortality from COVID-19 [12]. Optimal nutritional zinc [13] and selenium [14] status before exposure to the virus may reduce disease severity. Berger et al. suggests that perhaps the higher mortality rate from COVID-19 in Switzerland is at least partly due to suboptimal selenium and another micronutrient status in the population, resulting in suboptimal immune protection [6]. Reduced consumption of certain macro- and micro-nutrients is associated with an increased risk of contracting various infections. For example, magnesium intake is inversely related to the concentration of hs-CRP, IL-6, and TNF-α, due to the role of magnesium as a cofactor for many enzymes, and its low status may be associated with a decrease in various biochemical reactions [15]. Similarly, the effectiveness of the functioning of the immune system is related to the levels of essential trace elements, such as zinc, copper, and selenium, which are required as cofactors for various enzymes involved in antioxidant reactions [16,17,18]. A deficiency of certain elements can increase the risk of contracting COVID-19 [5]. The length of the disease, its form, and the severity of complications largely depend on the degree and duration of microelement deficiency [19]. Some of the above studies describe the current trace element status of patients with COVID-19, analyzing the relationship of individual trace element levels with symptom severity and disease progression [12]. Some studies focus on the initial dietary status of patients as, with COVID-19 [5,6,13,14,18,19] and with other infections [15,16,17]. However, most studies describing the trace element status of COVID-19 infections are observational studies; significant evidence of a change in elemental status in this disease in the form of systematic reviews with meta-analysis still needs to be presented.

When assessing the elemental status of a population, it is essential to remember that the deficiency of macro- and trace elements, as well as their excesses, may not be pronounced, forming a suboptimal elemental status, which, in turn, may vary in different populations groups [5]. Macro- and trace element deficiencies develop progressively over time, and subclinical manifestations occur long before the onset of clinical deficiency symptoms [20]. Elemental status depends on age, gender, nutritional status, physical activity level, health status, seasonal changes, and environmental pollution [21,22,23]. When assessing the elemental status of those who have recovered from COVID-19, we should remember that an active infection can lead to the loss of macro- and trace elements, such as calcium, zinc, and iron [19]. Multiple mineral deficiencies often occur together, such as low iron and zinc status [24].

Our study aimed to investigate the content of essential trace elements in the hair of unvaccinated residents of the Caspian region who recovered from COVID-19.

## 2. Materials and Methods

### 2.1. Participants

A cross-sectional study was carried out in the Caspian region on the territory of the Mangistau oblast, located in the southwest of Kazakhstan, east of the Caspian Sea, on the Mangyshlak plateau. The study was conducted in February 2021, before the start of mass vaccinations [25]. Permanent residents aged 18 to 60 entered the study. Exclusion criteria were: acute infectious, surgical, and traumatic diseases, chronic somatic diseases in the stage of decompensation, occupational diseases, metal implants (including amalgam fillings), vegetarian diet, intake of vitamin and mineral supplements, pregnancy, childbirth less than one year ago, and lactation. Detailed descriptions of the sample, recruitment procedures, material collection, and analysis procedures were described in our earlier publications [26,27].

Informed consent was obtained from each patient. The study was approved by the local ethics committee of the Marat Ospanov West Kazakhstan Medical University (meeting No. 5 on 13 May 2020) and accomplished following the principles of the Helsinki Declaration and subsequent amendments.

The study involved 260 residents of the Mangistau oblasts. The mean age of the participants was 40 (30; 49) years, and among them, 138 (53.1%) were males, and 102 (39.2%) participants out of the 260 reported having had a coronavirus infection (COVID-19). The mild course of COVID-19 was found in 42 (41.2%) participants, with moderate and severe severity in 60 (58.8%). The diagnosis and severity of COVID-19 were based on clinical signs and symptoms, laboratory data, R-graph results, and oxygen saturation of the clinical protocol [28]. The control group consisted of 158 patients without COVID-19.

### 2.2. Sample Collection and Analysis

Clean, stainless-steel scissors were used to cut hair samples from the back of the head. Only the proximal part of the hair was collected.

We estimated the concentrations of Co, Cr, Cu, Fe, I, Mn, Se, and Zn in the hair samples. Inductively coupled plasma mass spectrometry (ICP MS) on a NexION 300D mass spectrometer (Perkin Elmer, Shelton, CT, USA), equipped with an ESISC-2 DX4 automatic dispenser (Elemental Scientific Inc., Omaha, NE, USA), assessed the hair trace element composition.

Hair samples went through the washing and microwave digestion preparation processes. First, the hair strands were washed with acetone. Second, they were rinsed with deionized water and dried in air at a temperature of 60 °C. Third, the samples of biosubstrates were weighed and placed into chemically stable Teflon test tubes with concentrated nitric acid. Fourth, the samples were decomposed in a microwave “Berghof Speedwave 4” system (Berghof Products&Instruments, GmbH, Eningen, Germany) at 170–180 °C degrees for 20 min. Finally, solutions obtained during decomposition were cooled, equalized, and then put into test tubes, which were volumized to 15 mL with distilled, deionized water. For the chemical analysis, the final solution was used. The Universal Data Acquisition Standards Kit (Perkin Elmer Inc., Shelton, CT, USA) system was used for calibrating. Internal online standardization was performed using an yttrium-89 and rhodium-103 isotope solution obtained from Yttrium (Y) and Rhodium (Rh) Pure Single-Element Standard (Perkin Elmer Inc., Shelton, CT, USA). A certified human hair standard GBW09101, “Human hair,” issued by the Shanghai Institute of Nuclear Research (Shanghai, China), was used for quality control.

### 2.3. Statistical Analysis

The distribution of numeric data was assessed using the Kolmogorov–Smirnov and Shapiro–Wilk tests. These variables had a non-Gaussian distribution, and they were presented as the median (Me) and percentile (Q25; Q75). Qualitative variables were given as absolute values and percentages. The non-parametric Mann–Whitney U test was applied to assess trace element concentration differences in two independent groups [29].

The strength and direction of the relationship between trace elements’ content in hair and a history of COVID-19 infection were analyzed using linear regression analysis. For this analysis, all skew variables were log-transformed (Ln). In multiple linear regression analysis, the results were corrected by confounding factors (age, gender, body mass index (BMI), smoking, and residence category: urban/rural) [30].

All statistical analyses were performed using Statistica v.10 (StatSoft, Tulsa, OK, USA) and the SPPS v.25 Modeler (IBM) software.

## 3. Results

As can be seen from Table 1, the two groups with COVID-19 (+), *n* = 102, and COVID-19 (−), *n* = 158, did not differ in terms of place of living, BMI, and smoking habits. COVID-19 survivors were older and more were female.

The concentrations of Cr, Cu, Fe, I, Mn, and Zn in the hair of people who had coronavirus infections were lower than those who did not (Table 1).

Participants with COVID-19 were divided into two groups according to the severity of infection: *n* = 42 (41.2%) had mild COVID-19, and *n* = 60 (58.8%) had moderate or severe COVID-19. Table 2 shows the characteristics of the groups.

The results showed significantly higher levels of Cu in the hair of participants with moderate or severe COVID-19 than mild COVID-19 (Table 2). We performed a linear regression analysis to find an association between trace element levels in hair and prior coronavirus infection. The results of the analysis are presented in Table 3.

According to a simple model, the predictor variable for a history of coronavirus infection had a statistically significant effect on the content of Cr, Cu, Fe, and Mn in hair. At the same time, the regression coefficients for Co, I, Se, and Zn were insignificant. The regression coefficients Cr, Cu, Fe, and Mn showed that with the presence of a COVID-19 infection in a subject’s history, the content of these trace elements in the hair decreased. In multiple linear regression analysis, we included variables such as age, gender, BMI, smoking, and urban or rural residence, which may have affected hair trace element content. As can be seen from Table 3, the significance of the regression coefficients had not disappeared. We concluded that the impact of COVID-19 on the content of essential trace elements was stronger than the influence of these variables.

## 4. Discussion

We studied the trace element status for essential elements in residents of the Caspian region of Kazakhstan who had and did not have a COVID-19 infection from July–August 2020.

In terms of the concentration of essential elements in hair, a significant difference between the groups of surveyed residents of the Caspian region was established by the content of Fe, Cu, I, Zn, Mn, and Cr; the differences between the groups in terms of the content of Co and Se were not significant (Table 1).

The spread of the coronavirus infection has made it relevant to determine the factors and prerequisites for both infection and the form and severity of the disease. Among them, undoubtedly, was the status of some essential elements [3,5,7,20]. In the context of infection with SARS-CoV-2 and COVID-19, such essential trace elements as Fe, Cu, Zn, Mn, and Se and their roles in the formation of antiviral immunity were discussed. Since the beginning of the SARS-CoV-2 pandemic, many publications on this topic have been published. Fe, Cu, Zn, Mn, and Se deficiencies have been found to impair many aspects of innate and adaptive immunity and increase susceptibility to infections [1,6,10,18,31,32,33,34,35].

However, much of the research on the association of essential trace element status with the COVID-19 disease focused on the role of baseline trace element status in susceptibility to infection and its course. Only a negligible number of studies [36,37], including this study, focused on determining the trace element status after a COVID-19 infection.

A decrease in the concentration of the essential elements Fe, Cu, I, Zn, Mn, and Cr in the hair of the residents of the Caspian region who recovered from COVID-19 can be considered a consequence of the disease, which led to a change in the trace element status. At the same time, this suggests initially low (prior disease) essential element levels among those who recovered from the infection, which served as one of the factors in the development of the disease.

Iron deficiency is a significant public health problem and the world’s most common essential trace element deficiency [38]. Anemia and impaired iron homeostasis are joint in hospitalized patients with COVID-19 [39]. Iron takes part in the proliferation and maturation of immune cells and regulates the production of cytokines [18]. Iron deficiency is a crucial modulator of innate and adaptive immune responses [6] and increases the risk of contracting respiratory viral infections [5]. The decrease in iron levels in the hair of post-COVID-19 patients observed in this study (Table 1) may be a consequence of a decrease in iron absorption in the body during the development of an inflammatory process, which is implemented using hepcidin to limit the available iron pool for proliferating bacterial and viral infections and to reduce excessive oxidative stress [40,41].

Significant decreases in copper levels were found in the hair of patients who recovered from COVID-19 (Table 1). Copper plays an essential role in the innate immune response [18] and is associated with producing IL-2, antibodies, and T-cell proliferation [5]. It should be noted that even a slight Cu deficiency negatively affects the activity of T-lymphocytes and phagocytic cells [5,20]. The severity of the disease correlated with the ratio of zinc to copper in patients with COVID-19 [42]. In a study by Hackler et al., surviving COVID-19 patients showed a higher mean of Cu in serum concentrations than non-survivors [43]. The excess Cu content found in our study in patients with more severe COVID-19 compared to the mild form is consistent with Bego T et al.’s data (2022). In this study, when comparing the content of Cu in blood serum in groups of patients with clinical conditions of varying severity, an increase in Cu was found with a more severe condition [44].

According to Umarova et al. [26], the levels of copper in the hair of the inhabitants of the entire population of western Kazakhstan as a whole remains significantly low. However, speaking about the relatively low levels of copper in the hair of the inhabitants of this region, it must be remembered that the biogeochemical province of the zone of dry steppes, deserts, and semi-deserts, to which the territory of western Kazakhstan belongs, is characterized by a deficiency of copper [45]. In addition, a significant part of the population of this region, including residents of the Caspian region, lives in the area of active production and processing of gas and oil, which leads to the pollution of the living environment with toxic products, including toxic trace elements. Batyrova et al. described an imbalance in the status of the content of toxic trace elements such as aluminum (Al), arsenic (As), and mercury (Hg) in the hair of the inhabitants of the Caspian region [27]. Copper is in antagonistic interactions with some trace elements from the toxic and essential groups [46,47]. Some authors attribute the decrease in the levels of copper in biosubstrates in residents of the areas of industrial pollution to an increase in the concentration in the environment and in the body of the examined heavy metals, which belong to the group of toxic and copper antagonists—lead and cadmium [48], aluminum, arsenic, and mercury [27]. It is possible that the inhabitants of the Caspian Sea, who recovered from the COVID-19 infection, had initially relatively low levels of copper in their hair before the disease. A cross-sectional study with a single sampling does not answer this question and requires further work in this direction.

Just like most of the representatives of the group of essential elements, zinc demonstrates a significant decrease in the concentration in the hair of the residents of the Caspian region who have recovered from COVID-19, compared with a group of healthy individuals (*p* = 0.011) (Table 1). Zinc plays a vital role in the development and integrity of the innate and acquired (adaptive) immune systems at the molecular, cellular, and systemic levels. Zn is a central regulator of all immunological mediators, controlling their primary cellular functions, including deoxyribonucleic acid (DNA) replication, ribonucleic acid (RNA) transcription, cell division, and activation [49].

The development of a COVID-19 infection in a significant proportion of patients was accompanied by intestinal symptoms such as diarrhea, with damage to enterocytes, and malabsorption or impaired intestinal secretion, resulting in the malabsorption of Zn and Cu [50]. It has been proven that Zn-containing compounds can effectively inhibit the viral activity of SARS-CoV-2 [51,52,53]. Zinc inhibits SARS-CoV-2 RNA polymerase, and therefore, its ability to replicate [54]. Zinc deficiency weakens the body’s resistance to pathogens, and at the same time, increases the risk of an overactive immune response that can cause tissue damage. According to Wessels et al., severe zinc deficiency may predispose patients to the severe progression of COVID-19 [55]. A decrease in serum zinc in patients with COVID-19, along with a decrease in the Cu/Zn index, was established by Singh et al. [56]. Bagher et al. found that serum Zn levels were strongly associated with patient outcomes (*p* = 0.005) [37].

Thus, the zinc deficiency found in the first group of surveyed residents of western Kazakhstan may mean a potential risk factor for increased susceptibility to the COVID-19 pathogen or a decrease in its level in the body as a result of an infectious process.

When studying the role of essential trace elements in ensuring the normal functioning of the immune system and resistance to infections, researchers usually focus on describing the roles of Fe, Cu, Zn, and Se [56,57], and the roles of Mn, I, and Cr are less affected. However, several recent papers describe changes in Mn [58,59], I, and Cr [58,59,60] concentrations in patients with COVID-19 infections.

Manganese deficiency is rare; on the contrary, excessive exposure to this metal increases the risk of its accumulation in the body [61]. Mn is part of several metalloenzymes or acts as an enzyme activator [62]. Significant differences in the content of manganese in the hair between the groups of those who recovered from COVID-19 and healthy controls (*p* < 0.001) suggest that it is the infection that is the factor in reducing its content in the convalescent group (Table 1). In a study by Zeng et al., when determining the content of trace elements in whole blood, decreases in the levels of manganese were found in patients with COVID-19, in the severe form, compared with patients who had mild illnesses [59]. Conversely, urinary manganese concentrations measured throughout the illness were higher in patients with severe COVID-19 [63]. It was established that manganese deficiency interferes with antibody production [58]. The search for a means to counter the coronavirus infection makes evidence of a pronounced immunomodulatory and antiviral effect of Mn [64] very relevant. Evidence also suggests an impaired antibody production in response to Mn deficiency, highlighting its critical role in boosting immunity [62]. Thus, it can be assumed that Mn deficiency leads to impaired antiviral protection and greater vulnerability of these individuals to COVID-19 infection.

Of interest is the decrease in the concentration of chromium in the hair of patients with the coronavirus infection (Table 1), which was confirmed by regression analysis data (*p* < 0.001) (Table 2), as established in this study. The issue of determining the status of chromium is currently open and debatable. The state of Cr deficiency, due to its presence in the human body in relatively small amounts, is challenging to determine. It is believed that the concentrations of chromium in urine, hair, and biological fluids cannot reflect the actual status of the body for this trace element [4]. For example, in a study by Muhammad et al., no significant differences were found between plasma chromium levels in COVID-19 patients and healthy controls [60]. In a study by Zeng et al., patients with severe COVID-19 had significantly higher whole blood chromium levels compared to non-severe patients [59]. Urinary chromium concentrations were also higher in patients with severe COVID-19 [63].

The significant differences found in the content of iodine in the hair between the groups of those who recovered from COVID-19 and the group of healthy residents of the Caspian region of Kazakhstan (*p* = 0.048) (Table 1), however, were not confirmed by regression analysis data (Table 2). Nonetheless, the determination of iodine status seems to be very relevant, specifically for regions characterized by a decrease in the iodine content in the environment and food. It is iodine deficiency and the development of hypothyroidism associated with it that can be the factor that causes a decrease in the body’s immune characteristics [5]. The Caspian Sea of the Republic of Kazakhstan belongs to the regions in which the iodine content in the soil is reduced, and iodine deficiency conditions are pretty common [65,66]. Iodine may support the innate immune system to fight bacterial and viral infections [67,68]. Early in the COVID-19 pandemic, it was suggested that Japan’s relatively low COVID-19 mortality rate was due to high iodine intake, despite Japan having the oldest population in the world [69,70]. However, given that trace element status was determined in the present study after COVID-19, the relatively low iodine status in hair may be due to a previous disease, which, according to various authors, can lead to damage and dysfunction of the thyroid gland [71,72,73,74]. This issue undoubtedly requires further research in this direction.

The results of comparing the concentrations of the essential elements Co, Cu, Fe, I, Mn, Se, Zn, and Cr in the hair of western Kazakhstan residents who had and did not have COVID-19 were confirmed by multiple linear regression analysis. According to this analysis, the decrease in Cu, Fe, Mn, Zn, and Cr levels in the hair of study participants is associated with past infection with COVID-19 (Table 2). When conducting a regression analysis, it must be taken into account that the concentration of essential elements in hair depends not only on their food intake and the environment but also on many additional endogenous and exogenous factors. Multivariate regression analysis models, adjusted for confounding factors such as age, gender, BMI, smoking, and urban or rural residence, confirmed the above patterns described for Cu, Fe, Mn, Zn, and Cr in the unadjusted model (M0) (Table 2).

Our study had some limitations. First, a sample with a large number of study participants was required. Second, trace element status was influenced by dietary habits, which have not been studied. Third, trace element status was not assessed from before the COVID-19 disease. Consequently, well-designed cohort studies with large numbers of participants evaluating the impact of dietary intake on trace elements in hair are required. Despite this, the data obtained indicate a change in the content of essential trace elements in the hair of recovered, unvaccinated residents of the Caspian region of the Republic of Kazakhstan.

## 5. Conclusions

The data obtained indicate that a past COVID-19 infection affects the trace element status of the inhabitants of the Caspian region of Kazakhstan. After a COVID-19 infection, the contents of Cr, Cu, Fe, and Mn decrease, while the concentrations of Cu among those who have recovered are higher in those who have had COVID-19 in a more severe form. We believe that further in-depth research is required.

## Figures and Tables

**Table 1:** Descriptive characteristics of study participants.

Variable	All, *n* = 260	COVID-19 (+), *n* = 102 (39.2%)	COVID-19 (−), *n* = 158 (60.8%)	*p*
Age, years Me (Q1; Q3)	40.0 (30.0; 49.0)	42.0 (33.0; 52.0)	39.0 (29.0; 49.0)	0.045
Female, (*n*.%)	122 (46.9)	62 (60.8)	60 (38.0)	0.0003
Male, (*n*.%)	138 (53.1)	40 (39.2)	98 (62.0)	
Urban, (*n*.%)	64 (24.6)	30 (29.4)	34 (21.5)	0.045
Rural, (*n*.%)	196 (75.4)	72 (70.6)	124 (78.5)	
Smoking, (*n*.%)	46 (17.7)	13 (12.8)	33 (20.9)	0.093
BMI, кг/м^2^ Me(Q1; Q3)	40.0 (30.0; 49.0)	27.48 (23.88; 29.75)	25.89 (22.72; 29.38)	0.129
Co, mg/g Me (Q1; Q3)	0.015 (0.006; 0.036)	0.014 (0.006; 0.041)	0.016 (0.006; 0.034)	0.886
Cr, mg/g Me (Q1; Q3)	0.870 (0.838; 0.910)	0.862 (0.708; 0.897)	0.876 (0.843; 0.916)	0.010
Cu, mg/g Me (Q1; Q3)	8.249 (6.486; 9.879)	7.764 (5.831; 9.379)	8.550 (6.789; 10.301)	0.006
Fe, mg/g Me (Q1; Q3)	18.597 (13.602; 33.184)	16.055 (12.550; 24.981)	21.391 (14.587; 37.029)	0.0001
I, mg/g Me (Q1; Q3)	0.235 (0.149; 0.429)	0.209 (0.141; 0.340)	0.267 (0.156; 0.476)	0.048
Mn, mg/g Me (Q1; Q3)	0.320 (0.201; 0.640)	0.245 (0.172; 0.453)	0.370 (0.239; 0.789)	0.000009
Se, mg/g Me (Q1; Q3)	0.510 (0.433; 0.571)	0.497 (0.405; 0.554)	0.519 (0.440; 0.580)	0.110
Zn, mg/g Me (Q1; Q3)	191.171 (159.257; 238.513)	182.154 (151.364; 215.494)	199.462 (166.558; 247.600)	0.045

**Table 2:** Characteristics of study participants who had a coronavirus infection, depending on the severity of the disease.

Variable	COVID-19 Mild *n* = 42 (41.2%)	COVID-19 Moderate and Severe, *n* = 60 (58.8%)	*p*
Age, years Me (Q1; Q3)	41.0 (34.0; 52.0)	43.0 (32.5; 52.0)	0.656
Female, (*n*.%)	21 (50.0)	41 (68.3)	0.062
Male, (*n*.%)	21 (50.0)	19 (31.7)	
Urban, (*n*.%)	31 (73.81)	41 (68.3)	0.550
Rural, (*n*.%)	11 (26.19)	19 (31.7)	
Smoking, (*n*.%)	9 (21.4)	4 (6.7)	0.028
BMI, кг/м^2^ Me(Q1; Q3)	27.15 (23.54; 30.34)	27.66 (24.26; 29.74)	0.980
Co, mg/g Me (Q1; Q3)	0.011 (0.006; 0.036)	0.015 (0.006; 0.043)	0.420
Cr, mg/g Me (Q1; Q3)	0.865 (0.528; 0.891)	0.859 (0.742; 0.908)	0.395
Cu, mg/g Me (Q1; Q3)	6.739 (5.657; 8.444)	8.210 (6.327; 9.830)	0.024
Fe, mg/g Me (Q1; Q3)	16.253 (12.550; 21.438)	15.815 (12.383; 26.202)	0.736
I, mg/g Me (Q1; Q3)	0.235 (0.162; 0.346)	0.203 (0.140; 0.313)	0.428
Mn, mg/g Me (Q1; Q3)	0.240 (0.170; 0.329)	0.267(0.172; 0.543)	0.651
Se, mg/g Me (Q1; Q3)	0.497 (0.440; 0.576)	0.497 (0.392; 0.549)	0.357
Zn, mg/g Me (Q1; Q3)	182.654 (154.306; 215.895)	177.761 (147.691; 212.445)	0.529

**Table 3:** Assessment of crude and adjusted differences in essential trace element content of hair: results of multiple regression analysis.

Element	Model 0	95% CI	*p*	Model 1	95% CI	*p*	Model 2	95% CI	*p*	Model 3	95% CI	*p*	Model 4	95% CI	*p*
Co	0.930	0.681; 1.270	0.646	0.881	0.640; 1.213	0.435	0.902	0.649; 1.255	0.540	0.901	0.649; 1.252	0.535	0.958	0.698; 1.315	0.789
Cr	0.883	0.825; 0.946	<0.001	0.872	0.814; 0.935	<0.001	0.868	0.808; 0.933	<0.001	0.868	0.808; 0.932	<0.001	0.871	0.811; 0.936	<0.001
Cu	0.884	0.813; 0.963	0.005	0.889	0.815; 0.969	0.008	0.889	0.812; 0.971	0.010	0.888	0.812; 0.971	0.009	0.875	0.803; 0.955	0.003
Fe	0.733	0.629; 0.852	<0.001	0.748	0.641; 0.873	<0.001	0.742	0.634; 0.869	<0.001	0.742	0.634; 0.868	<0.001	0.745	0.636; 0.873	<0.001
I	0.865	0.675; 1.110	0.254	0.839	0.650; 1.084	0.180	0.832	0.639; 1.083	0.170	0.831	0.638; 1.082	0.169	0.827	0.634; 1.078	0.159
Mn	0.633	0.402; 0.519	<0.001	0.651	0.529; 0.802	<0.001	0.645	0.522; 0.799	<0.001	0.645	0.521; 0.797	<0.001	0.642	0.518; 0.795	<0.001
Se	0.960	0.858; 1.074	0.475	1.009	0.902; 1.129	0.869	1.016	0.905; 1.140	0.789	1.015	0.905; 1.140	0.793	1.029	0.918; 1.154	0.622
Zn	0.917	0.835; 1.005	0.064	0.945	0.863; 1.035	0.219	0.950	0.866; 1.042	0.275	0.950	0.866; 1.042	0.274	0.951	0.867; 1.044	0.291

Model 0: adjusted for COVID-19; Model 1: adjusted for age and gender; Model 2: as in Model 1, with the addition of BMI; Model 3: as in Model 2, with the addition of smoking; Model 4: as in Model 3, with the addition of urban/rural.

## Data Availability

Data are available from the corresponding author upon request.
